# A review of food poisoning caused by local food in Japan

**DOI:** 10.1002/jgf2.384

**Published:** 2020-10-14

**Authors:** Takashi Watari, Takayuki Tachibana, Azusa Okada, Kasumi Nishikawa, Kazuya Otsuki, Nobuhiro Nagai, Haruki Abe, Yasuhisa Nakano, Soshi Takagi, Yu Amano

**Affiliations:** ^1^ Postgraduate Clinical Training Center Shimane University Hospital Shimane Japan; ^2^ Faculty of Medicine Shimane University Shimane Japan

**Keywords:** food poisoning, Japanese local food, sashimi, sushi

## Abstract

Increasingly popular worldwide, Japanese cuisine includes several raw preparations such as sashimi and sushi; however, limited information on food poisoning from Japanese local food is available in English literature. Without appropriate knowledge, physicians may underdiagnose traveler's diarrhea among people returning from Japan. To provide accurate information to primary care physicians worldwide, we conducted a narrative review on food poisoning research published in Japanese and English over the past four years, considering the frequency and clinical importance of various presentations.

## Graphical abstract

A map of representative food poisoning caused by local food in Japan. The causes of food poisoning range from chemicals and natural toxins to pathogens such as bacteria and viruses, but can be broadly divided into bacterial, viral, parasitic, natural toxin, and five others. Of these, bacterial infection is the most common, followed by viral and parasitic infections in Japan.

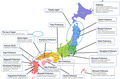

## 1 BACKGROUND

Japanese food culture is increasingly popular worldwide. Excluding fast food, Italy and Japan are the largest net exporters of local cuisine.^1^ The number of tourists entering Japan has also increased rapidly in recent years; annually, 31.88 million people visit to enjoy Japanese food culture.^2^ Japanese cuisine includes many examples of raw preparations such as sashimi and sushi; however, few English reports are available on the potential for food poisoning. This lack of knowledge may lead to physicians underdiagnosing traveler's diarrhea in individuals returning from Japan. Therefore, we conducted a review of food poisoning research published in Japanese and English over the past four years. We focused on data of clinical importance, such as case frequency.

## 2 METHODS

### 2.1 Data sources and search strategy

From October 2019 to February 2020, a literature search was performed across three online databases: MEDLINE (English), Google Scholar (English and Japanese), and ICHUSHI Web (Japanese), with the explode function when available and without language restriction. Based on information from the Ministry of Health, Labor and Welfare, we listed instances of Japanese‐cuisine‐derived food poisoning. In addition to the databases, our sources included a manual search of local healthcare center homepages, bibliographies of relevant articles, personal inquiries to experts, and certified physicians. The inclusion criteria of food poisoning in Japan we used are (a) content that is useful for foreign doctors’ practice, (b) pathogens are not common in other countries but are common in Japan, (c) diseases that are well known in Japan but little known abroad, and (d) new diseases that have become better known. At the same time, content on traveler's diarrhea, which is common knowledge in foreign countries, is omitted. Finally, we chose the eight clinically significant examples of Japanese local food poisoning (anisakiasis, ciguatera, tetramine poisoning, *Campylobacter jejuni/coli*, tetrodotoxin, hepatitis E infection (HEVi), *Kudoa septempuctata*, and mushroom poisoning) unfamiliar to physicians working outside Japan. Ethical approval was not required, as this study did not involve human subjects.

## 3 RESULTS

**FIGURE 1:**
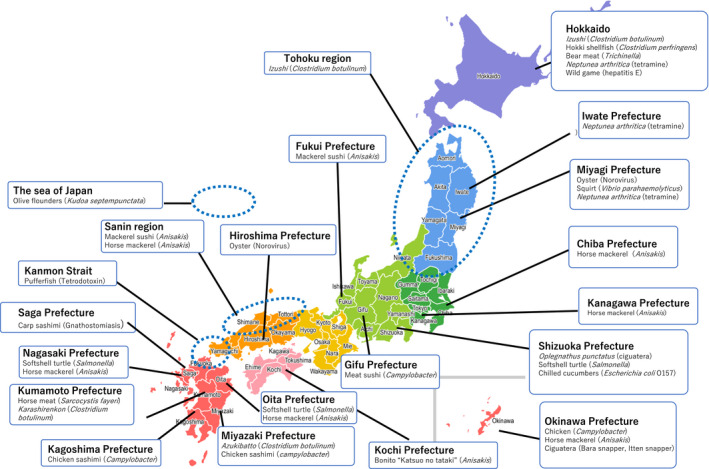
A map of representative food poisoning cases caused by local Japanese cuisine

We recorded at least 1000 cases of food poisoning per year in Japan from 2015 to 2018 (Table 1). This number excluded cases of suspected food poisoning. Figure 1 shows a map of Japan with representative types of food poisoning due to local cuisine.

**TABLE 1:** Food poisoning in Japan, 2015 to 2018

	2015	2016	2017	2018	Total
Bacteria	431	480	449	467	1827
*Salmonella* spp, nontyphoidal	24	31	35	18	108
*Staphylococcus aureus*	33	36	22	26	117
*Clostridium botulinum*	0	0	1	0	1
*Vibrio parahaemolyticus*	3	12	7	22	44
Enterohemorrhagic *Escherichia coli*	17	14	17	32	80
*Clostridium perfringens*	21	31	27	32	111
*Campylobacter*	318	339	320	319	1296
Other bacteria	15	17	20	18	70
Virus	485	356	221	265	1327
Norovirus	481	354	214	256	1305
(caused by oyster consumption)	(42)	(35)	(4)	(18)	(99)
Other viruses	4	2	7	9	22
Parasite	144	147	242	487	1020
*Kudoa septempunctata*	17	22	12	14	65
*Sarcocystis fayeri*	0	0	0	1	1
Anisakis	127	124	230	468	949
Other parasites	0	1	0	4	5
Natural toxin	96	109	60	61	326
Plant toxins	58	77	34	36	205
Mushroom	40	42	15	21	118
Grass	14	28	10	12	64
Potato	4	4	5	3	16
Other plant toxins	0	3	4	0	7
Animal toxins	38	32	26	25	121
Fish	6	8	4	2	20
Shell	3	6	3	9	21
Puffer	29	18	19	14	80
Other	46	47	42	50	185
Total	1202	1139	1014	1330	4685

The causes of food poisoning can be broadly divided into five categories: bacterial, viral, fungal, parasitic, and natural toxins. Of these, bacterial infection is the most common, followed by viral and parasitic infections. Among bacterial species, *Campylobacter jejuni* and *C coli* are the primary and most frequent (~300 cases/y) causes of food poisoning. Approximately 15 cases of food poisoning occur each year because of *Staphylococcus aureus* and enterohemorrhagic *Escherichia coli*. Among viral infections, norovirus food poisoning accounted for approximately 250 virus‐linked cases (>90%) annually. *Anisakis* is the dominant source of parasite‐linked food poisoning, causing over 100 outbreaks (>90%) annually. Recently designated food‐poisoning pathogen *Kudoa septenpunctata* constitutes approximately 10% of parasite‐caused cases. Finally, natural toxins derived from plants, fungi, and animals cause more than 60 cases of food poisoning annually. Pufferfish venom is the most common animal toxin, with approximately 10 cases per year, and shellfish poisoning generates around five cases per year. Mushrooms are the most common source of fungal/plant toxins, linked to approximately 30‐50 cases per year, while grasses are linked to ~10 cases per year. Across all sources of pathogen‐related food poisoning, food poisoning caused by *Norovirus* and *Campylobacter jejuni*/*coli* were the most common, followed by that caused by *Anisakis*.^2^, ^3^ Together, these four sources accounted for approximately 70% of all food poisoning cases. This review discusses, in great detail, the eight most significant causes of food poisoning from Japanese local food, ordered by the number of reported occurrences in our study.^2^, ^3^

## 4 PARTICULAR REMARKS

### 4.1 Campylobacter jejuni/coli

#### 4.1.1 Epidemiology

*Campylobacter jejuni/coli* are the most frequent cause of bacterial food poisoning in Japan. Annually, Japan sees approximately 300 cases and 2000 patients. Most infections occur from restaurants,^4^ and inappropriate handling of chicken causes nearly 90% of cases.^5^ Notably, raw meat, such as chicken sashimi and raw liver, is consumed in the unique Japanese food culture and can be a cause of food poisoning. Of note, the consumption of raw beef liver and pork meat is prohibited by law; however, some restaurants still provide these “delicacies.” Demographic analysis indicates that *Campylobacter* poisoning has two peaks, in early childhood (1‐4 year old) and in young adults (15‐34 year old). Men are more frequently infected, and adults tend to exhibit stronger symptoms.^6^

#### 4.1.2 Etiology

Chickens harbor *Campylobacter* spp. in their intestinal tract, and contamination of chicken meat from improper cleaning occurs in meat‐processing plants. Therefore, even packed chicken meat at a grocery store may be contaminated.^7^, ^8^ *Campylobacter* spp. can invade human epithelial cells, triggering apoptosis to cause cell damage and diarrhea.^9^

#### 4.1.3 Clinical manifestation

The incubation period for *Campylobacter* spp. infection is usually 24‐72 hours, and sometimes more than 1 week. Major symptoms are watery diarrhea (occasionally frequent and bloody), abdominal pain, and fever. Similar to other parasitic infections such as yersiniosis and salmonellosis, *Campylobacter* can mimic appendicitis when patients experience right lower abdominal pain.^10^

#### 4.1.4 Diagnosis and treatment

Identification of *Campylobacter* in stool samples is the gold standard for diagnosis. However, stool cultures require several days of preparation. Hence, symptoms are often resolved by the time test results are returned. Another occasionally used diagnosis method by veteran doctors is examining Gram stain smears.^11^

Antimicrobial therapy is generally not recommended, and the mainstay treatment is supportive care with fluid electrolyte infusion. Exceptional situations are hematochezia, high fever, prolonged symptomatic duration (1 week or longer), pregnancy, and HIV infection or immunocompromised status.^12^ Quinolones (eg, ciprofloxacin) were once considered the standard treatment in exceptional cases, but quinolone resistance is rising globally. Therefore, macrolides (eg, azithromycin) are now the first‐line therapy for reducing illness duration.^11^, ^12^, ^13^

#### 4.1.5 Prognosis

Almost all patients will recover in a few days, but some may develop late complications such as Guillain‐Barré syndrome (GBS) and reactive arthritis in the delayed phase.^14^, ^15^ The incidence rate of GBS is estimated at one in 1000 symptomatic patients and can occur even in the context of asymptomatic infections.^16^ Besides, the incidence of Campylobacter reactive arthritis is around 1% to 5% of infected patients.^17^

### 4.2 Anisakiasis

#### 4.2.1 Epidemiology

*Anisakis* is a genus of nematodes that parasitize marine mammals; fish such as mackerel, horse mackerel, and sardines; and other aquatic organisms such as squid. Japan is among the highest‐risk countries for anisakiasis because people frequently consume raw fish. From 2013 to 2015, most cases of *Anisakis* food poisoning were caused by marinated mackerel fillets called “*shime‐saba*,*”* rather than sashimi (traditional Japanese form of raw fish consumption).^18^

#### 4.2.2 Etiology

Anisakid larvae invade gastric and gut mucosa after ingestion. Anisakiasis involves two mechanisms: an allergic reaction and direct invasion of host intestinal walls. The former cases show symptoms ranging from urticaria and angioedema to life‐threatening anaphylactic shock. The latter cases often result in formation of eosinophilic granuloma or perforations.^19^

#### 4.2.3 Clinical manifestation

General manifestations of anisakiasis include gastric, intestinal, extraintestinal, and allergic symptoms. In Japan, 95% of anisakiasis is gastric.^19^ Gastric anisakiasis causes sudden abdominal pain at 1‐12 hours postingestion, along with nausea, vomiting, and slight fever. Intestinal anisakiasis is characterized by intermittent or continuous abdominal pain for 5‐7 days after ingestion of larva. Symptoms of extragastric anisakiasis differ depending on parasite location.^20^

#### 4.2.4 Diagnosis and treatment

Patients with acute gastric anisakiasis who consumed raw fish in the previous 12 hours are recommended to undergo gastrointestinal endoscopy. Treatment for gastric anisakiasis involves detection of larva through endoscopy and using biopsy forceps.^21^ No therapeutic medication for intestinal anisakiasis currently exists, but albendazole treatment appears to have some therapeutic properties in patients with intestinal obstruction.^22^ When a patient has allergic symptoms with or without endoscopic observation of larva, elevation of total or specific immunoglobulin E at 1 month post‐allergic reaction can help diagnose gastric allergic anisakiasis.^23^

#### 4.2.5 Prognosis

Vomiting and removal of larva with forceps improve symptoms. Anisakid larva cannot survive in the human intestine for more than a few days. Therefore, even if endoscopy is not performed, supportive treatment is sufficient in most cases.

### 4.3 Tetrodotoxin (pufferfish poisoning)

#### 4.3.1 Epidemiology

Japan is one of the few countries that eat pufferfish and among the biggest consumers, so most poisoning cases are from Japan. Annually, 30‐50 casualties (including lethal cases) are reported. This number increases from autumn to winter.^4^

#### 4.3.2 Etiology

Tetrodotoxin (TTX) is the most well‐known causative agent of pufferfish poisoning, but the fish also contain other toxins (eg, saxitoxin). Pufferfish does not directly produce TTX. Instead, *Pseudomonas* or *Vibrio* in the ocean synthesize TTX, which then accumulates in the fish's ovary or liver.^24^, ^25^ TTX is extremely toxic; even a very low concentration can affect the voltage‐gated sodium channels on the surfaces of vascular smooth muscles, skeletal muscles, and neurons, inhibiting their depolarization. However, TTX generally does not affect cardiac muscles.^26^

#### 4.3.3 Clinical manifestation

Eating pufferfish ovary or liver is prohibited because it almost always contains TTX. The prohibition extends to muscle or skin in some species that contain TTX in those parts.^25^, ^27^ Individuals handling pufferfish must have valid licenses, and most cases occur at home in individuals without a license. Heating or washing cannot eliminate or inactivate TTX.

#### 4.3.4 Diagnosis and treatment

Without commercial tests, almost all cases are diagnosed via clinical reasoning. Definitive diagnosis is made through TXX identification from urinary or blood specimens of patients or from their leftover meals. Symptoms occur rapidly, minutes to hours after ingestion. Typical clinical manifestations are paresthesia of the face or around the mouth and paralysis of extremities. Some patients develop respiratory muscle paralysis, which can be lethal. There are four grades of severity.^28^

As no antidote or specific treatments are known, symptomatic treatment is the mainstay. If poisoning is reported within 1 hour postonset, gastric lavage or activated carbon ingestion are recommended. Extracellular fluid or atropine administration is considered for bradycardia or hypotension. In the event of respiratory failure, artificial ventilation and intubation are used.^28^ The known cure is to wait for the patient's body to excrete the toxin.

#### 4.3.5 Prognosis

Most symptoms occur in 6‐8 hours; hence, re‐exacerbation after 24 hours is very rare. Prognosis is favorable if the patient survives the first 24 hours and has intensive care.

### 4.4 Kudoa septempunctata

#### 4.4.1 Epidemiology

First reported in 2010, *Kudoa septempunctata* is a novel Myxosporean approximately 10 µm in size, with 6‐7 valves and polar capsules. This flower‐shaped organism parasitizes as a cyst in olive flounder muscles.^29^, ^30^ Initially considered nontoxigenic for humans, *K septempunctata* has recently been linked to food poisoning. In Japan, the parasite causes approximately 10 cases of foodborne illnesses annually,^31^ with most incidents traced back to hatchery flounders from Korea. However, recent research has shown that native fish can also be parasitized.^32^

#### 4.4.2 Etiology

Most patients with *Kudoa* poisoning develop diarrhea through the following possible manner, although detailed mechanisms are unknown.^29^, ^33^ After ingestion, *Kudoa* releases the sporoplasm, which invades gut epithelial cells. This loosens tight junctions between epithelial cells, causing fluid accumulation in the intestinal lumen and eventually diarrhea.

#### 4.4.3 Clinical manifestation

Predominant symptoms are diarrhea, abdominal pain, and emesis. Some patients are febrile. The average incubation period is 4 hours.^34^, ^35^ Because a large number of *Kudoa* in the intestines is necessary for poisoning, eating parasitized olive flounder does not necessarily cause illness.

#### 4.4.4 Diagnosis and treatment

Diagnosis usually occurs using polymerase chain reaction or microscopic identification of *Kudoa* in patient samples.^36^, ^37^ However, these tests are not implemented in clinical settings, and other laboratory tests have low efficacy. Therefore, close questioning of whether patients ate olive flounders is essential.^31^ *Kudoa* symptoms are transient and resolve in several days because the parasite cannot survive in the human body. However, supportive care (infusion fluid, antiemesis, and antifebrile treatment) may be needed if symptoms are severe. Prevention measures include freezing (−20°C for ≥4 hours or −80°C for ≥2 hours) or cooking (75°C for ≥5 minutes).

#### 4.4.5 Prognosis

*Kudoa* poisoning is a self‐limited disease with no known fatalities.

### 4.5 Toxic mushroom (*Lampteromyces japonicus*)

#### 4.5.1 Epidemiology

Japan experiences approximately 30 cases of mushroom poisoning annually, with most cases occurring between September and November.^38^, ^39^, ^40^

#### 4.5.2 Etiology

Each poisonous mushroom species contains a different toxin, leading to a wide variety of symptoms. In Japan, “tsukiyotake (*Lampteromyces japonicus*)” and its specific toxin illudin S causes around half the cases. Some experiments show that illudin S causes cell damage through reacting to the sulfate of cysteine or glutathione in gastrointestinal epithelial cells.^39^ Heating or washing cannot inactivate illudin S.^39^

#### 4.5.3 Clinical manifestation

Although clinical symptoms of mushroom poisoning vary widely, tsukiyotake poisoning causes gastrointestinal symptoms such as nausea, vomiting, and diarrhea within 30 minutes to several hours postingestion.

#### 4.5.4 Diagnosis and treatment

Detailed history taking and contact with the regional poisoning center should be done. Since the cases caused by commercially available mushrooms are extremely rare, clarifying route of acquisition is essential for diagnosis.^38^, ^40^ Specific treatments are unavailable, so only symptomatic treatments are used.

#### 4.5.5 Prognosis

Serious cases are rare, with reported mortality being about 0.4% between 2015 and 2018.^38^

### 4.6 Tetramine poisoning

#### 4.6.1 Epidemiology

Several members of genus *Neptunea* are toxic, with grain shellfish among the most recognized. Sold under the name of “bai‐gai,” or “tsubu‐gai” in Japan, the whelk is linked to several cases of poisoning (<30) annually, with no reported fatalities.^41^ According to 2018‐2019 statistical data, Yamaguchi and Hokkaido prefectures are the main consignors for bai‐gai and tsubu‐gai, respectively. Whelks are on the market throughout the year, but shipments peak from June to July.^42^ In Japan, reported *Neptunea* poisoning cases from 2014 to 2018 tended to increase during April and November in Fukushima and Tokyo prefectures.^43^ *Neptunea* are distributed worldwide; researchers from Korea, Denmark, Scotland, and Canada have also reported whelk poisoning.^44^

#### 4.6.2 Etiology

*Neptunea* poisoning is due to tetramine, a chemical produced in the salivary gland of many whelk species.^44^ Tetramine exhibits neuromuscular blockade properties, interacts with the autonomous nervous system, and stimulates parasympathetic nerves.^45^

#### 4.6.3 Clinical manifestation

Ophthalmalgia, headache, dizziness, nausea, vomiting, diplopia, and somnolence occur 30‐60 minutes after ingestion.

#### 4.6.4 Diagnosis and treatment

Removal of whelk salivary glands is the most effective way to prevent poisoning. Diagnosis requires history taking for whelk consumption without salivary gland removal and the postingestion time course. Tetramine is a heat‐stable substance, and cooking (boiling and slow thawing) can cause diffusion from salivary glands to other tissues.^46^

The toxic dose threshold of tetramine is not well established. Average tetramine concentration per whelk differs across studies,^47^ and some studies have even demonstrated between‐season differences in a single individual.^48^ Thus, the total number of ingested whelks may help determine poisoning, whereas location and season may not inform diagnosis.

#### 4.6.5 Prognosis

Only supportive treatment is needed, and symptoms tend to resolve in a few hours.

### 4.7 Ciguatera

#### 4.7.1 Epidemiology

The genus *Gambierdiscus* includes dinoflagellates that produce ciguatoxin. Biological concentrations allow high levels of ciguatoxin to accumulate in marine organisms. Ciguatera fish poisoning (CFP) occurs when a patient consumes fish contaminated with high ciguatoxin concentrations. Yellow‐edged lyretail (*Variola louti*), One‐spot snapper (*Lutjanus monostigma*), and Two‐spot red snapper (*Lutjanus bohar*) are the source of most cases. Although global warming has increased the number of CFP cases in the southern region of Japan (Kyushu area), the vast majority occur in Okinawa (the southernmost prefecture). The annual number of CFP cases is 15 (including suspected cases).^49^

#### 4.7.2 Etiology

Ciguatoxin lowers the threshold of voltage‐gated sodium channels in neural cell membranes and causes depolarization, releasing Gamma‐aminobutyric acid and dopamine. Patients then experience a variety of neurological symptoms.^50^ Stable under heating or freezing, ciguatoxin cannot be eliminated through cooking, and the only avoidance method is to prevent ingestion of ciguatoxin‐contaminated fish.^51^

#### 4.7.3 Clinical manifestation

Typical symptoms such as nausea, vomiting, abdominal pain, or diarrhea develop within 6‐24 hours after consumption. In some cases, fatigue, headache, and weakness may last for several months. Cardiac symptoms (acute bradycardia and hypotension) are common, with occasional dyspnea. Neurological symptoms include pain or numbness in the extremities, paresthesia (hands, toes, around the mouth), pruritis, myalgia, and arthralgia. The most characterized symptom of ciguatera is temperature‐related dysesthesia (so‐called “dry ice sensation”), where cold surfaces are perceived as hot. In most cases, this symptom becomes prominent after gastrointestinal symptoms within the first few days.^50^ Oh et al^52^ have also reported reversible cerebellar dysfunction in CFP.

#### 4.7.4 Diagnosis and treatment

A reliable detection test is currently unavailable. Thus, history taking for consumption of at‐risk fish, gastrointestinal symptoms, and specific neurological symptoms are the most important steps for diagnosis. Differential diagnoses include shellfish poisoning, mackerel poisoning, pufferfish poisoning, enterovirus infection, bacteremia, organophosphorus poisoning, arsenic poisoning, multiple sclerosis, eosinophilic meningitis, botulism, and Guillain‐Barré syndrome.^50^ Seeking other patients with similar symptoms also aids diagnosis. Symptomatic treatment remains the mainstay without a well‐established specific treatment. Some researchers have shown beneficial effects of mannitol infusion, but this efficacy is controversial.^53^, ^54^

#### 4.7.5 Prognosis

In approximately 50% of CFP cases, patients experience persistent neurological symptoms for two weeks or more.^55^ Complete recovery from neurological symptoms sometimes requires several months.^49^ Mortality is <0.5%.^55^ In some reports, alcohol, fish, caffeine, nuts, chicken, and pork can trigger recurrent symptoms.^56^

### 4.8 Hepatitis E infection (HEVi)

#### 4.8.1 Epidemiology

Gibier or game in Japan typically comes from wild boar or deer, which are hunted year‐round. Recent cases of venison intoxication in Japan have provided important evidence that hepatitis E virus infection (HEVi) is zoonotic and likely a source of food poisoning.^57^, ^58^ However, other routes of infection are possible, including uncooked pork, contaminated water, and shellfish.^57^, ^58^

#### 4.8.2 Etiology

Hepatitis E virus (HEV) is a positive‐sense single‐stranded RNA virus that causes acute hepatitis, but the specific mechanism is unclear.^59^, ^60^, ^61^, ^62^, ^63^, ^64^, ^65^ As it is noncytopathic, hepatocyte destruction likely occurs from host immunological reaction. Human HEV has four genotypes.^57^ Genotypes 3 and 4 are the causative pathogens of food poisoning in Japan, and genotype 4 is unevenly distributed in Hokkaido prefecture.

#### 4.8.3 Clinical manifestation

Viral incubation lasts 2‐9 weeks. Fever, fatigue, nausea, emesis, abdominal pain, and jaundice are the chief manifestations. In addition to typical hepatitis, neurological dysfunction and renal failure can occur as extrahepatic symptoms.^63^ In Japanese clinical settings, most HEVi cases are identified upon medical examination. Asymptomatic patients are often misdiagnosed with drug‐induced hepatitis or not diagnosed at all. Therefore, identifying HEVi as a differential diagnosis is the essential first step. Fulminant hepatitis is very rare, but pregnant or elderly patients are at high risk. Such high‐risk patients should be treated carefully.^59^, ^60^, ^61^, ^62^, ^63^, ^64^, ^65^, ^66^, ^67^, ^68^, ^69^ Although genotype 4 HEVi is a high‐risk agent, recent studies have showed that host factors are more important than viral genotype when determining risk.^62^, ^63^, ^64^, ^65^, ^66^, ^67^, ^68^, ^69^

#### 4.8.4 Diagnosis and treatment

The IgA‐HEV antibody is important for facilitating early diagnosis. Monitoring the patients’ liver function is essential because HEV does not have a specific treatment.

#### 4.8.5 Prognosis

Most cases of acute HEV are self‐limited and have good prognosis. Transition to chronic hepatitis is rare, but has been reported.^69^

## 5 DISCUSSION

When examining patients with acute gastroenteritis, general physicians always consider food poisoning due to *Campylobacter*, *Norovirus*, and *Anisakis* because they account for the majority of cases. However, food poisoning from less common sources occur annually, and awareness of these sources is also necessary. Most food poisoning incidents occur among tourists who enjoy eating local specialties without knowing that they may contain pathogens. Food poisoning also occurs when consumers eat carelessly, or without knowing that self‐collected ingredients might contain natural poisons.

Here, we reviewed causative agents of food poisoning in Japanese local cuisine, with the aim of helping physicians diagnose patients with symptoms specific to food poisoning. We especially wish to highlight that cases involving ciguatera, tetramine poisoning, pufferfish poisoning, HEV, and *K septempunctata* depend on regional consumption. Thus, even Japanese primary care physicians have not encountered many such cases and may not recognize or suspect the cause of food poisoning.

To the best of our knowledge, this is the first review on food poisoning caused by Japanese local cuisine. Although patients can spontaneously recover after being infected by many of the described causative agents, some sources have neurotoxins that can be lethal. Our survey has several limitations. First, we have only included confirmed food poisoning cases (~1000) from 2015 to 2018 and physicians are not obligated to report Campylobacter enteritis and norovirus infections in Japan; therefore, the exact number of food poisoning cases remains unknown. Second, we omitted general traveler's diarrhea, but we did so because most physicians can provide a differential diagnosis of the aforementioned diseases to travelers with diarrhea returning from Japan. However, here we provided relevant data so that they can include Japanese local food as a potential source of infection.

## 6 CONCLUSION

Epidemiological reports of Japanese local food poisoning in English are sparse despite an increase in the number of overseas travelers to Japan. Although not an exhaustive list, here we reviewed, for the first time in English, Japanese foodborne illnesses and their causative agents. Our goal was to facilitate food poisoning diagnosis in travelers to Japan who returned with gastroenteritis. We hope that this review will aid further investigation and documentation of Japanese foodborne illnesses.

## CONFLICT OF INTEREST

The authors have stated explicitly that there are no conflicts of interest in connection with this article.
